# Relationship between Monoclonal Gammopathy of undetermined significance and multiple myeloma via online database analysis

**DOI:** 10.12669/pjms.39.3.7206

**Published:** 2023

**Authors:** Weiguo Lu, Jiaming Liu, Man Luo, Mingfeng Xiao

**Affiliations:** 1 Weiguo Lu, Clinical Laboratory, The First Affiliated Hospital, Guangzhou University of Chinese Medicine, Guangzhou 510405, China; 2 Jiaming Liu, The Second Clinical Medical College, Guangzhou University of Chinese Medicine, Guangzhou 510405, China; 3 Man Luo, Hematology Department, The First Affiliated Hospital, Guangzhou University of Chinese Medicine, Guangzhou 510405, China; 4 Mingfeng Xiao, Clinical Laboratory, The First Affiliated Hospital, Guangzhou University of Chinese Medicine, Guangzhou 510405, China

**Keywords:** Multiple Myeloma, Monoclonal Gammopathy of Undetermined Significance, Bioinformatics, PI3K/AKT/mTOR, Inflammation, Immune dysfunction

## Abstract

**Objective:**

To explore the relationship between Monoclonal Gammopathy of undetermined significance (MGUS) and Multiple Myeloma (MM) based on bioinformatics methods.

**Methods:**

In this study, we conducted bioinformatics to identify genes associated with MGUS and MM using the PubMed pubmed2ensemble (http://pubmed2ensembl.ls.manchester. ac.uk/) until 2021. Gene ontology function was used to label overlapping genes, and Kyoto Encyclopedia of Genes and Genomes analysis was used to identify enriched pathways. The cluster-1 genes obtained from Cytoscape were analyzed by Comparative Toxicogenomics Database (CTD, http://ctdbase.org/) and then used to screen candidate drugs using the DSigDB database (https://amp.pharm.mssm.edu/Enrichr/).

**Results:**

In total, 227 genes were common to both MGUS and MM. These genes were significantly associated with cytokine-cytokine receptor interaction and the PI3K-Akt signaling pathway. The protein-protein interaction network revealed that TNF, IL-1B, IL-6, CSF2, CXCL8, and IL-10 were among the core genes of MM. Finally, eight candidate drugs showed maximum interaction with core genes, which could potentially prevent MGUS from progressing to MM.

**Conclusion:**

The progression of MGUS to MM is driven by aberrant cytokine secretion, which leads to inflammation immune dysfunction, and dysregulation of the PI3K/AKT/mTOR signaling pathway.

## INTRODUCTION

Multiple myeloma (MM) is a malignant proliferative disease of plasma cells that usually affects the elderly population, which is characterized by heterogeneity in the clinical course.^1^ The common clinical manifestations of MM include hypercalcemia (C), renal damage (R), anemia (A), and bone damage (B), collectively known as CRAB symptoms. In the past 20 years, novel targeted drugs have significantly improved the prognosis of MM, although the disease remains incurable.^2^ In Europe and the Americas, the incidence rate of MM has increased annually and is currently 6/100,000.^3^ MM can be divided into three stages: monoclonal gammopathy of undetermined significance (MGUS), asymptomatic or smoldering myeloma (SMM), and asymptomatic or active myeloma (AMM). MGUS is a precancerous plasma cell disease that can persist for almost 10-20 years before it progresses to MM, which underscores the clinical significance of diagnosing MGUS in a timely manner and preventing its transformation to MM in order to prolong patient survival.

Studies have shown that age >60 years, male sex, African-American ethnicity, family history of MM, antigen activation, obesity, infection, immune disorders, and downregulation of cd27/tnfrsf7 are risk factors for B-cell transformation to MGUS. Osteoporosis, neuropathy and thrombophlebitis are also related to the progression of MGUS.^4^ In addition, MGUS was more prevalent among men than women (4% vs. 2.7%), and 5.3% of the affected individuals were older than 70 years. The incidence among African Americans was twice that in Caucasians, whereas those of Japanese ethnicity had lower incidence rates compared to Caucasians. First-degree relatives of patients are at increased risk for MGUS, indicating the involvement of genetic factors. The transformation of MGUS into MM is associated with IgH translocation, hyperdiploidy, and cyclin D disorder. This is accompanied by changes in the bone marrow microenvironment, such as increased production of IL-6, IGF-1, and matrix metalloproteinases; impaired T cell function; enhanced osteoclast function; and inhibition of osteoblasts. However, the exact molecular mechanisms underlying the evolution of MM remain unclear. We applied bioinformatics methods to identify common genes between MGUS and MM and functionally annotated them through GO and KEGG pathway analyses. A protein-protein interaction (PPI) network was constructed to identify the core gene clusters of MGUS and MM. In addition, we screened candidate drugs for MGUS and MM based on core genes.

## METHODS

Genes related to MGUS and MM were retrieved from Pubmed2ensemble (http://pubmed2ensembl.ls.manchester. ac.uk/). Input our target disease ‘MGUS’ and ‘multiple myeloma’ separately, to receive the gene set of relevant terms, pick ‘retrieve up to 100,000 document IDs’ and then ‘Homo sapiens genes (GRCh37)’. The MGUS and MM gene sets were categorized into list one and list two using the online tool Venny 2.1 (https://bioinfogp.cnb.csic.es/tools/venny/). GOTERM_BP_FAT, GOTERM_CC_FAT, and GOTERM_MF_FAT was downloaded after GO and KEGG_PATHWAY analyses. BP, CC, and MF are abbreviations of biological process, cellular component, and molecular function, respectively.

The intersecting genes between MGUS and MM were analyzed using STRING (https://string-db.org) with the following settings: multiple proteins, species: Homo sapiens, and minimum required interaction score:0.900.^5^ The disconnected nodes in the network are hidden. The PPI network was downloaded as a TSV file and imported to Cytoscape. Functional modules were analyzed using the MCODE plugin, and core genes were identified.

Comparative Toxicogenomics Database (CTD, http://ctdbase.org/), which integrates chemical-gene/protein, chemical-disease, and gene-disease relationships,^6^ was used to analyze core genes and MM risk.

### Identification of candidate drugs:

Based on the core genes for MGUS and MM, candidate drugs were screened from the Drug Signatures Database (DSigDB, https://ngdc.cncb.ac.cn/), which consists of 22527 gene sets.^7^ The Enrichr platform (https://amp.pharm.mssm.edu/Enrichr/) was used to analyze and visualize the data.^8^

### Ethical Compliance:

Not applicable. This article does not involve human and animal experiments. This article extracts relevant information based on public databases for text mining and data analysis, which is not within the scope of ethical review.

## RESULTS

**Fig.1:**
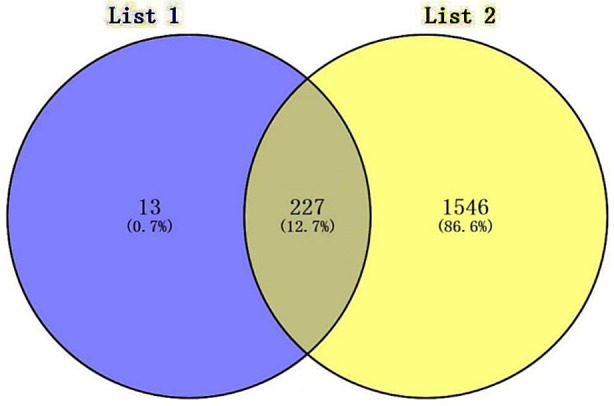
Common differentially expressed genes representation through a Venn diagram.

We identified 240 MGUS-related and 1773 MM-related genes, of which 227 genes were common to both (Fig.1).

**Fig.2:**
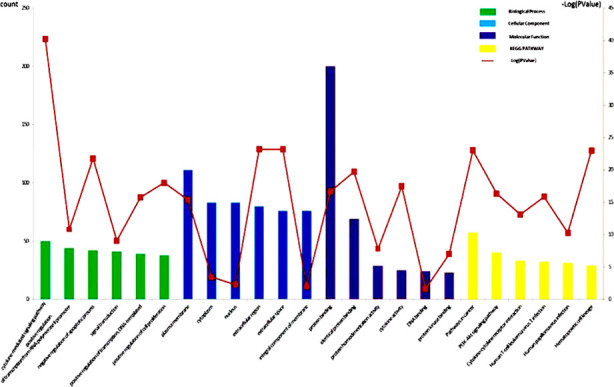
The top-6 of GO enrichment and KEGG pathway enrichment. Count means how many genes fall on the biological function/signal pathway. -Log (P-Value) means calculate the -Log10 value of P value for easy display in the figure.

Based on the overlapping genes, the top-6 biological processes, cellular component terms, and molecular function terms were listed in Fig.2. The top-6 KEGG pathways associated with these genes contained PI3K-Akt signaling pathway, cytokine-cytokine receptor interaction and so on (Fig.2).

**Fig.3:**
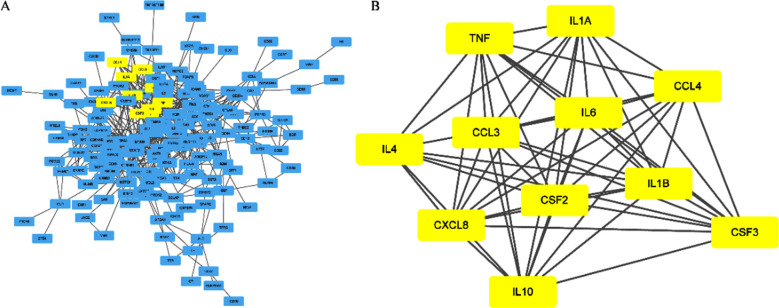
PPI network. (A): The 227 common genes. (B) Network of cluster-1 gene.

The PPI network consisted of 166 points and 645 edges (Fig.3A). The most prominent gene module was cluster-1, which included 11 core genes (Fig.3B). Core genes contain TNF, IL-1B, IL-1A, CCL4, CCL3, IL-6, CSF2, CSF3, CXCL8, IL-10, and IL-4.

**Fig.4:**
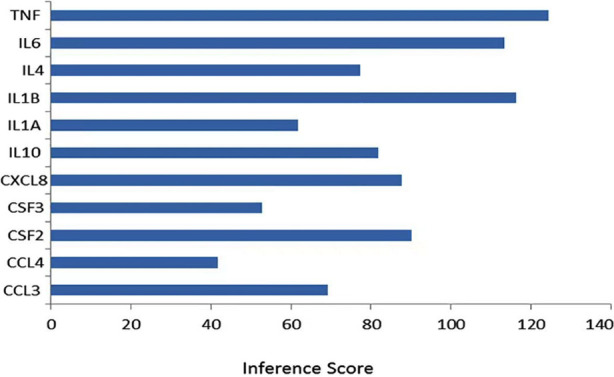
The inference scores between the core genes and MM progression.

The connection between core genes and MM progression was evaluated utilizing CTD data in terms of inference scores. As shown in Fig.4, TNF, IL-1B, IL-6, CSF2, CXCL8, IL-10, and IL-4 had the highest scores.

The Enrichr platform was used to identify candidate drugs for MM based on the 11 core genes. Using data from the DSigDB database, we identified titanium dioxide CTD 00000489, dinoprostone CTD 00007049, beclomethasone CTD 00005468, mustard gas CTD 00006356, ionomycin CTD 00007090, phencyclidine CTD 00005881, gemcitabine CTD 00002382 and phorbol 12-myristate 13-acetate CTD 00006852 as the candidate drugs (Table-I) that most genes interacted with. As these signature drugs were detected for the core genes, they could potentially target MGUS and MM.

**Table-I:** Suggested top drug compounds for the MM.

Name of drugs	P-value	Adjusted P-value	Genes
Titanium dioxide CTD 00000489	1.97E-24	3.51E-21	IL4;IL10;IL1A;IL6;CSF3;CSF2;CXCL8;IL1B;CCL4;CCL3;TNF
Dinoprostone CTD 00007049	8.12E-22	7.22E-19	IL4;IL10;IL1A;IL6;CSF2;CXCL8;IL1B;CCL4;CCL3;TNF
Beclomethasone CTD 00005468	1.66E-21	9.83E-19	IL4;IL10;IL6;CSF2;CXCL8;IL1B;CCL4;CCL3;TNF
Mustard gas CTD 00006356	1.73E-19	7.55E-17	IL10;IL1A;IL6;CSF3;CSF2;CXCL8;IL1B;CCL4;CCL3;TNF
Ionomycin CTD 00007090	2.12E-19	7.55E-17	IL4;IL10;IL6;CSF2;CXCL8;IL1B;CCL4;CCL3;TNF
Phencyclidine CTD 00005881	3.03E-19	8.97E-17	IL10;IL1A;IL6;CSF3;CSF2;CXCL8;IL1B;CCL4;CCL3;TNF
Gemcitabine CTD 00002382	4.50E-19	1.14E-16	IL4;IL10;IL1A;IL6;CSF3;CSF2;CXCL8;IL1B;CCL3;TNF
Phorbol 12-myristate 13-acetate CTD 00006852	1.46E-18	3.24E-16	IL4;IL10;IL1A;IL6;CSF3;CSF2;CXCL8;IL1B;CCL4;CCL3;TNF

## DISCUSSION

MGUS is an asymptomatic disease characterized by the involvement of monoclonal immunoglobulins (M protein) and precedes plasma cell malignancies such as multiple myeloma (MM). Landgren et al.^9^ hypothesized that all MM cases originate from MGUS given that the incidence rates of MGUS 2, 3, 4, 5, 6, 7, and 8+ years before MM diagnosis were 100%, 98.3%, 97.9%, 94.6%, 100.0%, 93.3%, and 82.4%, respectively. Furthermore, early obesity may increase the overall risk of MM. Recently, studies have increasingly shown that early intervention and prevention are the most effective means of managing MM, for it is an incurable disease that can lead to irreversible organ damage. Recent studies have shown that no reliable biomarkers were existed currently to predict the progression of MGUS to MM. Therefore, elucidation of the molecular mechanisms underlying disease progression is essential to identify patients with MGUS who are at high risk for progression to MM, as well as potential therapeutic targets.

In this study, we identified 227 genes that were differentially expressed in MGUS and MM patients relative to healthy controls using two datasets. A PPI network was then constructed, which revealed that CSF2, TNF, IL-6, CCL4, IL-4, IL-1B, CXCL8, CSF3, IL-10, CCL3, and IL-1A, of which TNF, IL-1B, IL-6, CSF2, CXCL8, IL-10, and IL-4 had the highest scores. Thus, aberrant proliferation and differentiation of plasma cells in MM can be attributed to a dysregulated cytokine profile.

TNF-α is a pleiotropic cytokine that mediates inflammatory responses and induces myeloma cells to produce high IL-6 levels. Mehtap et al.^10^ compared the serum cytokine profiles of 44 newly diagnosed MM patients and healthy controls and found that TNF-α levels were elevated in the sera of MM patients relative to controls. Furthermore, TNFα levels were higher in D-S stage III patients than in Stage-I and II patients, indicating that it is closely associated with MM. In addition, TNFα can induce anemia in patients with MM by directly inhibiting the formation and differentiation of red blood cells, reducing sensitivity to erythropoietin (EPO), and promoting IL-6 secretion by myeloma cells.

IL1β is a pro-inflammatory cytokine^11^ that is primarily secreted by monocytes and macrophages and encoded by the IL1B gene. It mediates acute and chronic inflammation, which culminates in the production of TNF-α and free radicals.^12^ IL-1β is upregulated in breast cancer, colon cancer, melanoma, and lung cancer^13^, although little is known about its role in the occurrence and development of MGUS and MM.

IL-6 is a key cytokine involved in plasma cell differentiation and proliferation of myeloma cells.^14^ The serum activity of IL-6 and levels of its soluble receptor (sIL-6R) are closely associated with myeloma progression and disease stage.^15^ The lower secretion of IL-4 in MM is closely related to elevated IL-6, which not only promotes B cell differentiation and proliferation of malignant plasma cells but also induces the generation of acute phase proteins (APPs), including C-reactive protein (CRP) and amyloid A. In fact, serum IL-6 activity and CRP concentration are positively correlated in MM patients. IL-10 drives the proliferation and differentiation of activated B cells and has recently proved to promote the proliferation of primary myeloma cells, although it does not induce plasma cell differentiation or immunoglobulin secretion. Furthermore, antibodies targeting IL-6 or IL-6R could not block IL-10-induced proliferation, indicating that IL-6 is not a determinant of IL-10 function. By contrast, IL-10 inhibited the secretion of endogenous IL-6. Despite its purported role as a growth factor in myeloma, the role of IL-10 in MM remains to be elucidated.

Granulocyte-macrophage colony-stimulating factor (GM-CSF, also known as CSF2) and granulocyte colony-stimulating factor (G-CSF, also known as CSF3) bind to their specific receptors on hematopoietic progenitor cells in the bone marrow and promote the maturation and survival of granulocytes, monocytes, macrophages, and T cells.^16^ In gliomas, GM-CSF and G-CSF may promote angiogenesis in the tumor microenvironment by activating the STAT-3 transcription factor or upregulating VEGF/VEGFR, increasing inflammatory cell infiltration, and thus promoting tumor progression.^17^ These cytokines also induce an immunosuppressive microenvironment in melanomas by expanding and stimulating myeloid-derived suppressor cells (MDSCs) in tumors, which dampens the antitumor response and thus promotes early growth and metastasis.^18^ Furthermore, increased expression levels of CSF2 and CSF3 promote the growth, and differentiation of various tumor cells, resulting in shorter survival and rapid metastasis.^19^

CSF3R is composed of two identical gp130 signal transduction chains that share a high degree of homology with gp130 in IL-6R. Both CSF3 and IL-6 activate nuclear factor IL-6 (NF-IL-6) by synergistically acting on the gp130 signaling pathway. CSF3 promoted the proliferation of myeloma cells, although it did not affect the expression of IL-6 and IL-6R, indicating that IL-6 does not mediate the proliferative effects of CSF3. However, IL-6 and IL-6R antibodies can block CSF3-induced myeloma cell proliferation. Interestingly, CSF2 does not directly act as a growth factor in myeloma, but instead sensitizes cells to IL-6. In addition, IL-3 and IL-5 share a common signal transduction chain, KH97, with CSF2, which can increase the sensitivity of myeloma cells to these cytokines. Furthermore, IL-3 can significantly upregulate the high-affinity receptor, IL-6, in myeloma cells. CXCL8 is a chemokine of the CXC family that recruits and activates neutrophils and other granulocytes, thus initiating the inflammatory response. Tumor cells secrete high levels of CXCL8 during metastasis.^20^

The PI3K/AKT/mTOR signal transduction pathway acts as an essential part in tumor development and progression, which is frequently dysregulated in MM, pancreatic cancer, gastrointestinal stromal tumors, etc. The activated PI3K/AKT signal transduction pathway promotes tumor cell migration, proliferation, and drug resistance via its downstream effectors and is therefore a potential anti-tumor therapeutic target. In fact, targeted blocking of this pathway, with or without the inhibition of other pathways, can trigger apoptosis of MM cells and has been shown to reduce bone degeneration in MM patients.^21^

We screened several candidate drugs for MM and MGUS based on 11 core genes using the DSigDB database, of which the top eight significant drugs were titanium dioxide CTD 00000489, dinoprostone CTD 0000704, beclomethasone CTD 00005468, mustard gas CTD 00006356, ionomycin CTD 00007090, phencyclidine CTD 00005881, gemcitabine CTD 00002382, and phorbol 12-myristate 13-acetate CTD. Gemcitabine is a pyrimidine nucleoside analog that induces apoptosis and inhibits the proliferation of MM cells. It has shown encouraging results against various types of NHL.^22^ Nabhan et al.^23^ further showed that gemcitabine can induce apoptosis in MM cells via the mitochondrial caspase cascade independent of IL-6. Similarly, Krett et al.^24^ discovered that it significantly inhibited the growth of MM cell lines in a dose-and time-dependent mode. These candidate drugs will have to be validated through in vitro studies in future clinical trials.

Most of the MGUS patients can only be monitored through regular review, which does not prevent them from developing to MM. Based on the literature and our research, we summarizes that inflammation and immune dysfunction based on PI3K/AKT/mTOR signaling pathway play an important role in the development of MGUS to MM and screened some candidate drugs, which provides an important idea to prevent the development of MGUS to MM by improving the patient’s immune function and inflammation. We will confirm our view through a series of basic and clinical experiments. Perhaps in the near future, we can reverse the development of MGUS to MM.

### Limitations of the study:

It is a bioinformatics article and has not been included in clinical or basic experiments that can prove its clinical applicability.

## CONCLUSION

Based on our findings, we can surmise that the progression of MGUS to MM is driven by aberrant cytokine secretion, which leads to inflammation immune dysfunction, and dysregulation of the PI3K/AKT/mTOR signaling pathway. Therefore, exploring the pathways underlying inflammation and immune dysfunction can offer new insights into the evolution of MM and possible therapeutic targets. In addition, the role of the PI3K/AKT/mTOR signaling pathway and other signaling pathways involved in the above process need to be studied further. A greater understanding of these mechanisms can help identify novel therapeutic targets and improve patient prognosis.

### Author`s Contribution

**WL** contributed to the conception, design, manuscript writing, data analysis, and interpretation of methodology. **JL** contributed to the provision of the study materials and manuscript writing, collection, and assembly of data. **ML** contributed to revised the manuscript. All authors contributed to the manuscript revision and read and approved the submitted version.

**MX** contributed to the conception, design, manuscript writing, collection, and assembly of the data conceptualization. He is also responsible and accountable for the accuracy or integrity of the work.
